# The development of sexual stage malaria gametocytes in a Wave Bioreactor

**DOI:** 10.1186/s13071-017-2155-z

**Published:** 2017-05-02

**Authors:** Corine G. Demanga, Jenny W. L. Eng, Donald L. Gardiner, Alison Roth, Alice Butterworth, John H. Adams, Katharine R. Trenholme, John P. Dalton

**Affiliations:** 1 Institute of Parasitology, McGill University, 21111 Lakeshore Road, Sainte-Anne-de-Bellevue, Québec, H9X 3 V9 Canada; 2 Malaria Biology Laboratory, QIMR Berghofer Medical Research Institute, 300 Herston Rd, Herston, Brisbane, Australia; 3 School of Medicine, University of Queensland, St Lucia, 4072 QLD Australia; 4 Department of Global Health, College of Public Health, University of South Florida, Tampa, 33612 FL USA; 5 School of Biomolecular and Physical Sciences, Griffith University, Nathan, 4111 QLD Australia; 6 School of Biological Sciences, Medical Biology Centre, Queen’s University of Belfast, 97 Lisburn Road, BT9 7BL Northern Ireland, UK

**Keywords:** *Plasmodium falciparum*, Gametocytes, In vitro culture, Wave Bioreactor, Malaria, Drug discovery, Vaccines

## Abstract

**Background:**

Blocking malaria gametocyte development in RBCs or their fertilization in the mosquito gut can prevent infection of the mosquito vector and passage of disease to the human host. A ‘transmission blocking’ strategy is a component of future malaria control. However, the lack of robust culture systems for producing large amounts of *Plasmodium falciparum* gametocytes has limited our understanding of sexual-stage malaria biology and made vaccine or chemotherapeutic discoveries more difficult.

**Methods:**

The Wave Bioreactor^TM^ 20/50 EHT culture system was used to develop a convenient and low-maintenance protocol for inducing commitment of *P. falciparum* parasites to gametocytogenesis. Culture conditions were optimised to obtain mature stage V gametocytes within 2 weeks in a large-scale culture of up to a 1 l.

**Results:**

We report a simple method for the induction of gametocytogenesis with N-acetylglucosamine (10 mM) within a Wave Bioreactor. By maintaining the culture for 14–16 days as many as 100 million gametocytes (stage V) were produced in a 1 l culture. Gametocytes isolated using magnetic activated cell sorting (MACS) columns were frozen in aliquots for storage. These were revitalised by thawing and shown to retain their ability to exflagellate and infect mosquitoes (*Anopheles stephansi*).

**Conclusions:**

The production of gametocytes in the Wave Bioreactor under GMP-compliant conditions will not only facilitate cellular, developmental and molecular studies of gametocytes, but also the high-throughput screening for new anti-malarial drugs and, possibly, the development of whole-cell gametocyte or sporozoite-based vaccines.

**Electronic supplementary material:**

The online version of this article (doi:10.1186/s13071-017-2155-z) contains supplementary material, which is available to authorized users.

## Background

According to the World Malaria Report 2014 there are an estimated 3.3 billion people at risk of malaria. In 2013, there were between 124 and 283 million cases of malaria and 367–755,000 deaths. Malaria is caused by protozoan parasites of the genus *Plasmodium* and among the five species that cause malaria in humans *P. falciparum* is the deadliest. Greater than 90% of malaria deaths occur in sub-Saharan Africa, predominantly in children below the age of five, where this species is most prevalent [1].

The life-cycle of *P. falciparum* parasites is complex and occurs within two hosts, the human and the female *Anopheles* mosquito [2]. In the human host, the parasites grow and multiply asexually within red blood cells (RBCs), destroying these when they emerge to invade new cells. As the number of infected RBCs increase, some parasites develop into sexual forms, the micro-(male) and macro-(female) gametocytes. Gametocytes develop through five distinct stages, with only mature stage V parasites able to undergo sexual reproduction when ingested by a feeding mosquito [3–5]. The microgametes and macrogametes of *P. falciparum* emerge from the ingested RBCs in the mosquito gut where fertilization occurs and results in the formation of a motile ookinete. The ookinete develops into an oocyst containing sporozoites that subsequently migrate to the mosquito salivary glands where they become fully mature after about 10 days. These mature sporozoites infect the next human host when the mosquito takes a blood meal [2, 5, 6].

Inhibiting gametocyte development in RBCs or preventing their fertilization in the mosquito gut can prevent infection of the vector and, therefore, passage of disease to the human host. To achieve the ambitious goal of malaria eradication it is envisaged that the strategy of ‘transmission blocking’ will have a vital role to play [7–10]. This approach would reduce or interrupt the spread of malaria disease in endemic regions and will be employed alongside vector control and case management by chemotherapy host. The Malaria Eradication Research Agenda (malERA) initiative has identified the development of new safe and effective drugs that block the infectivity of mature *P. falciparum* gametocytes as a priority research area. However, identification of compounds with activity against late stage *P falciparum* gametocytes is challenging as this stage is relatively metabolically inert and in vitro culture methods to produce gametocytes are much less straightforward than for asexual stage parasites. The 8-aminoquinoline primaquine is currently the only licensed antimalarial drug effective against late stage gametocytes, but known side effects of primaquine limit its usefulness [6, 8, 11, 12]. In addition to the discovery of new drugs that kill gametocytes, there has been a long-term interest in developing a vaccine that specifically targets the sexual stages and interrupts malaria transmission [7–10].

While studies on the asexual stages of *P falciparum* were greatly facilitated by the development of an in vitro culture system [13] the lack of a robust culture system for sexual stages has limited our understanding of *P falciparum* gametocyte biology and made studies with these stages much more difficult. The most commonly used methods for gametocyte production start with cultures of asexual stage parasites, which are then “induced’ to differentiate into early stage gametocytes that develop to maturity in a stable culture period of 10–14 days [14]. The mechanism of commitment to sexual development of *P. falciparum* is poorly understood, although this switch is suggested to occur in the generation preceding gametocytogenesis [15] and are dependent on genetic, epigenetic and transcriptional factors [6]. The stimuli leading to commitment act on the merozoite and/or the early ring stage parasite [6, 15–17] and factors such as host immunity and presence of antimalarial drugs may increase gametocytogenesis in vivo, while erythrocyte lysates, host immune sera, hypoxanthine, conditioned medium, high asexual parasite density and cAMP inducing pathway have been exploited, with variable results, to trigger or enhance gametocytogenesis in vitro [3, 5, 6, 15–20].

Several methodologies for small-scale gametocyte culture have been published but regardless of the method employed, the production of gametocytes in vitro is technically challenging, time consuming, costly, and lacks stability and reproducibility [4, 21–24]. Even in parasite lines with relatively high conversion rates, gametocytes usually represent less that 1% of the parasitized cells within a given culture [6, 15] and, consequently, the amount of gametocyte material that can be isolated from in vitro systems is not conducive to large or high-throughput studies. Recently, Delves et al. [25] and Duffy et al. [26] reported detailed protocols for the routine culture of gametocytes in volumes between 0.2 and 200 ml.

We described the large-scale production of asexual blood stage *P. falciparum* cultures in the Wave Bioreactor^TM^ 20/50 EHT system [27], which is a closed sterile plastic bioreactor that rocks on a heated platform. Here, we report use of the Wave Bioreactor^TM^ for the development of a convenient and low-maintenance protocol for producing gametocytes and subsequent maintenance of these to obtain mature stage V gametocytes in a large-scale culture of up to a 1 l. As many as 100 million mature *P. falciparum* gametocytes can be produced and purified from each culture and these can be cryopreserved and resurrected for in vitro studies. The resurrected gametocytes were shown to successfully infect mosquitoes.

## Methods

### Asexual parasite culture

The *Plasmodium falciparum* strains 3D7 and FCR3 were selected for this study. The incomplete culture medium was prepared from RPMI 1640 powder with L-Glutamine (Gibco/Invitrogen, Montreal, Canada) at 10.5 g/l, buffered with 24 mM sodium bicarbonate (NaHCO_3_), 35 mM HEPES and Hypoxanthine (Sigma-Aldrich, Montreal, Canada). Prior to use, Albumax-I (0.5% w/v) and 25 μM of gentamycin (Gibco/Invitrogen) were added to the medium to give complete culture medium. Medium used for experiments performed at QIMRB was identical to that described above with the exception that Albumax-1 was replaced by 10% human serum. Fresh human erythrocytes preserved in CPDA-1 (citrate-phosphate-dextrose-adeninine) anticoagulant solution were obtained once a month from Interstate Blood Bank, Memphis, Tennessee, USA, or from the Australian Red Cross Blood Service, Brisbane, Australia. They were washed twice with 10 volumes of RPMI-1640 and suspended at 50% (vol./vol.) with complete RPMI medium.

Parasites were routinely cultured in T150 flasks with vented caps (Corning Life Science, Montreal, Canada) in a volume of 50 ml at 4% haematocrit either for seeding the bioreactor or for growth comparisons with the bioreactor. Medium change was performed daily or twice per day (for high parasitemia) and flasks were kept in a HERACell 240i incubator (Thermo Scientific, Montreal, Canada) regulated with a gaseous environment of 3% O_2_, 5% CO_2_ and 92% N. To synchronize the cultures, sorbitol treatment was performed on parasites ring stage and repeated after 48 h [28, 29].

### The Wave Bioreactor system for *P. falciparum* blood stage culture

The Wave Bioreactor^TM^ 20/50 EHT system (GE Healthcare, Montreal, Canada) was used for this study. This system is composed of a presterilized transparent inflatable plastic bag, or Cellbag, which is the disposable cultivation chamber. Each Cellbag has integrated inlet and outlet air filters and ports for adding medium and extracting samples (Additional file 1: Figure S1), for this study a 2 l Cellbag, recommended for cell culture volumes of 0.1 to 1 l was used. The Cellbag is placed on a platform that sits on a rocking unit, which is designed to inflate and rock the bioreactor for rapid gas transfer. Using a touch-screen connected to the rocking unit, the motion (speed and angle) and temperature of the platform and bioreactor is tightly controlled. The volume and rate of gas entering the bioreactor is also regulated. The platform of the Wave Bioreactor^TM^ 20/50 EHT system contains two separate heating panels, allowing single or dual operation, i.e. one or two 2 l bioreactor chambers can be run at a given time. Several parameters within the bioreactor chamber can be controlled remotely by a WavePOD^TM^ controller, including dissolved oxygen (disO_2_), pH, and O_2_/CO_2_ gas mixing. Although this controller is an optional unit, we employed it in the present study to monitor disO_2_ during culture of *Plasmodium* in the Cellbag. We did not use the WavePOD^TM^ controller to create the gas mix within the bioreactor; instead, an analyzed gas mix of 3% O_2_, 5% CO_2_ and 92% N was directly fed from a cylinder into the bioreactor via the rocking unit. The protocol for culture of asexual *P. falciparum* blood stage in the Wave Bioreactor is described in Additional file 1: Figure S1.

**Fig. 1:**
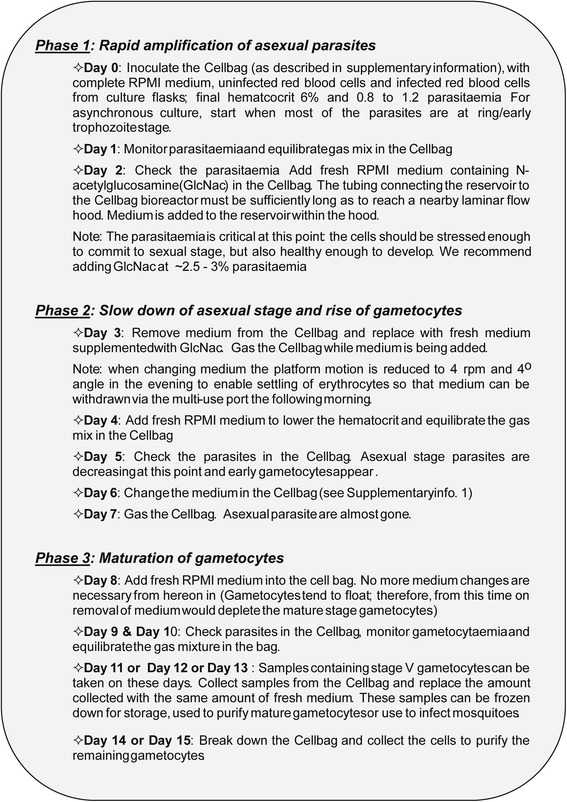
Procedure for the large-scale production of *P. falciparum* gametocytes in the Wave Bioreactor

To optimise gametocyte production in the Wave Bioreactor, several experiments with both synchronous or asynchronous blood stage cultures were performed independently at the McGill University in Canada and QIMR Berghofer Medical Research Institute (QIMRB) in Australia. Therefore, we have developed a straightforward protocol to produce *P. falciparum* gametocytes in the Wave Bioreactor that is described in Fig. 1 approximately 10–15 days before initiation of gametocyte production in the Wave Bioreactor, *P. falciparum* aliquots were thawed and cultured as described. Schizont enrichment by flotation on 1% (wt./vol.) gelatin (Sigma-Aldrich) was performed to replace old RBCs in the culture with fresh ones [28]. Parasites where synchronized as described for inoculation in the Cellbag [28, 29]. The starting volume in the Cellbag was between 350 and 500 ml.

Once induction of gametocytogenesis had occurred N-acetylglucosamine (GlcNAc, Sigma-Aldrich) was added to the Cellbag at a final concentration of 10 mM to prevent further proliferation of asexual stage parasites. Aliquots of GlcNAc stock solution were prepared at 1 M, stored at -20 °C and thawed immediately prior to use. Culture samples (2–5 ml) were harvested daily from the Cellbag through the multi-use port via a 10 ml luer-lock syringe and using to monitor parasitemia and gametocytaemia, as well as to measure glucose consumption, lactate production and pH levels. Glucose and lactate measurement was performed at the Montreal General Hospital, Canada, with the blood gas analyzer ABL825 Flex (Radiometer, Diamond Diagnostic-USA). Parasite cell counts and assessment of specific developmental stages were performed with light microscope on Giemsa-stained thin smears. The parasitemia and gametocytaemia were determined over the examination of between 2000 and 12,000 RBCs. The morphological criteria of stage II-V gametocytes described elsewhere [3] were used to evaluate the maturity of gametocytes on Giemsa-stained thin smears. The sex of mature stage V gametocytes was differentiated by morphology as depicted by Mitri et al. [30].

### Gametocyte purification

During the production of gametocytes in the Wave Bioreactor, mature gametocytes can be purified over four consecutive days starting at day 10 of the culture. Sixteen hours prior to the purification, the speed and the angle of the rocking unit were adjusted to 2 rpm and 3°, respectively, to allow the RBCs to settle in the Cellbag. Since gametocyte infected RBCs tend to float as the parasites mature, they were collected by gently removing the supernatant via the multi-used port. From each 1 l culture bag, 200–400 ml of supernatant was removed each day and replaced with fresh complete RPMI medium. The bioreactor was then returned to 8 rpm through an angle of 6° to allow mixing of the remaining culture. The isolation of gametocytes from the cells harvested in the supernatant was performed by magnet-activated cell sorting (MACS) as previously described [4]. Gametocyte numbers were quantitated by haemocytometer.

### Cryopreservation of gametocytes and procedure for thawing

Given the substantial number of gametocytes that can be produced in the Wave Bioreactor, we used the freezing method for gametocytes developed by Keister and Kaslow [31] and modified by Peatey et al. [14]. Cell samples (200–600 ml) were collected during 3–4 days from the Cellbag, and replaced by fresh complete RPMI medium as described above. The cell pellet obtained by centrifugation of the samples at 700× *g* for 10 min was washed with 10 volume of RPMI. The glycerin-based solution Glycerolyte 57 (Fenwall Inc., Lake Zurich, Il, USA) was added to the cell pellet to yield a final concentration of 85% (v/v) in two steps. First, 1/5 of the pellet volume of Glycerolyte 57 was added drop wise to the cells and after about 2 min at room temperature, the remaining volume was added and the cell suspension was distributed in cryotubes. The cryotubes were placed at -80 °C for 24 h and then transferred to liquid nitrogen for storage.

To thaw the gametocytes produced in the Wave Bioreactor, frozen aliquots removed from liquid nitrogen were thawed in a water bath at 37 °C for approximately 1 min. The contents of vials were transferred into a centrifuge tube and a warm solution of 12% NaCl equal to 0.2 volume of the thawed cells was added drop by drop with gentle shaking. The parasite suspension was let stand for 1–2 min at room temperature and a warmed solution of 1.6% NaCl equal to 10 volumes of the original vials contents was added slowly to the cell suspension. The cell suspension was centrifuged at 700× *g* for 4 min, and the pellet was suspended in a 0.9% NaCl warmed solution equal to 10 vol. of the original vial contents, then centrifuged as before. Samples of the pellet were taken to prepare a thin smear for Giemsa-stain or for exflagellation test and the remaining was suspended in complete RPMI for time development study.

### Assessment of gametocytes viability after freezing

Giemsa-stained thin smears made with samples of frozen cells prepared at different time after thawing, were examined by microscopy for morphological evaluation of the different gametocytes stages. For the in vitro exflagellation assay, the exflagellation-inducing medium containing 100 μM of xanthurenic acid (XA, Sigma-Aldrich) was prepared as described by Bhattacharyya and Kumar [32]. All the components of the exflagellation test where kept at room temperature. Cell samples obtained after thawing, or freshly collected from the Bioreactor were centrifuged as above and the pellet was suspended at 25% hematocrit in exflagellation-inducing medium. To examine exflagellation, an aliquot of the cell suspension was collected after 10–15 min and transferred on a coverslip attached with silicone on cell chamber. The sample was diluted with exflagellation-inducing medium, to allow visualization of single cells, and analysed by confocal differential interference microscopy (DIC).

### Assessment gametocyte infectivity

The cryopreserved gametocytes were shipped on dry ice to the University of South Florida where the mosquito membrane feeding assays (MFA) were performed. After rapid thawing by the procedure described above using graded salt solutions gametocytes were added to a warmed mixture of human serum and RBCs at a ratio of 1:1.6. Fifty female *Anopheles stephensi* mosquitoes starved for 4 h were fed with small volumes of gametocytes samples (500–1000 μl) through the Hemotek 5 W1 membrane feeding system (Discovery Workshop, England) at 37 °C, or at room temperature to prevent rapid desiccation, although mosquitoes fed poorly on the unheated samples. Then the mosquitoes were transferred in chamber set at 26 °C and 80% relative humidity and feed with 10% sugar solution on alternate days. The infected mosquitoes were dissected to determine midgut oocysts on day 8–12 and salivary gland sporozoites on days 16–20 post-blood meal.

## Results

### Protocol design for the large-scale culture of *P. falciparum* gametocytes in the Wave Bioreactor

In our original protocol developed for the large-scale culture of asexual stages in a Wave Bioreactor we reported that the Cellbag in which the parasites were cultured must be removed daily from the heated rocking platform and taken to a laminar flow hood for medium change [27]. To simplify and streamline this culture process a reservoir bag filled with medium was connected to the Cellbag via the extended inlet tubing creating a completely closed system (Additional file 1: Figure S1). Medium can be removed from the Cellbag via the multi-port unit while it remains *in situ* on the platform and fresh medium added by opening the connection between the reservoir and the Cellbag. In this modified protocol, set-up of a 1 l culture is completed in less than 2 h and, thereafter, the Cellbag is not removed from the heated rocking platform for the entire process. This prevents parasites being exposed to temperature fluctuations that occurred in our earlier protocol.

Using the protocol set out in Additional file 1: Figure S1, asexual *P. falciparum* parasites, *3D7* and *FCR3*, grow very favourably (Additional file 2: Figure S2a). Measurements of glucose consumption in the parasite cultures showed that this nutrient is maintained within optimal limits for growth (8 to 10 mM) (Additional file 2: Figure S2b). Furthermore, lactate build-up, with the consequent reduction of pH, is controlled so that these do not reach levels that are detrimental to the parasite, i.e. lactate above 12 mM and pH below 7 (Additional file 2: Figure S2b, c). The ability to control conditions within the parameters optimal for *P. falciparum* parasite growth by replenishing medium daily ensured that a parasitemia of 5–6% was routinely obtained over four to 5 days in 1 l cultures.

### *P. falciparum* gametocyte induction in the Wave Bioreactor

Our long-term culture of *P. falciparum* within the Cellbag bioreactor paved the way for the development of a protocol for the reliable large-scale production of the sexual stages. In our large-scale gametocyte production protocol (Fig. 1), sexual stage induction occurs during Phase 1 and Phase 2 leading to development of mature Stage V gametocytes ready for mosquito infection in phase 3. Gametocyte development is susceptible to temperature fluctuations that significantly deviate from normal body temperature and adversely affecting their ability to reach maturity. Thus, the addition of the medium reservoir to the system so that there is no need to remove the Cellbag from the heated platform was greatly beneficial (the temperature within the Cellbag never dropped below 35 °C).

**Fig. 2:**
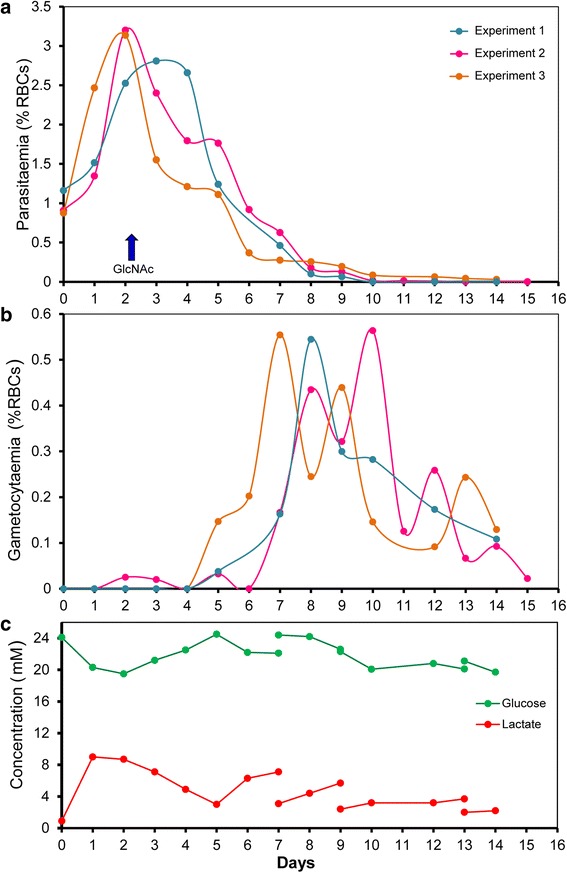
Time course for the development of *P. falciparum* gametocytes in the Wave Bioreactor starting from asynchronous blood stage culture. **a** Proliferation of asexual parasites and gametocyte induction in three representative experiments, *arrow* represents the time of addition of GlcNAc. **b** Evolution of gametocytaemia for the corresponding experiments presented in** a**. **c** Measurements of glucose and lactate in the medium during the production of gametocytes in the Wave Bioreactor

During the proliferation of *P. falciparum* asexual blood stage parasites, only a small proportion will commit and differentiate into gametocytes [4, 6, 15]. The rate of commitment to gametocytogenesis is enhanced in response to stress, such as occurs when parasite density can increase dramatically during in vitro culture. Therefore, to enhance production of gametocytes in the Wave Bioreactor, asexual stage cultures were established at a starting parasitemia of between 0.8 and 1.1% and allowed to multiply rapidly such that at day 2 the parasitaemia was almost triple the starting parasitaemia (Fig. 2a). During this initial period, the culture medium in the Wave Bioreactor was intentionally not changed to stress the parasites and induce gametocytogenesis [19, 33]. As alluded to above, when asexual parasites multiply, they rapidly deplete the glucose in the medium and produce lactic acid (Fig. 2c), which results in a rapid drop of pH to levels that are detrimental to gametocyte development*.* For this reason, we used N-acetylglucosamine (GlcNac) to halt the growth the asexual parasites before their density became too high [34].

**Fig. 3:**
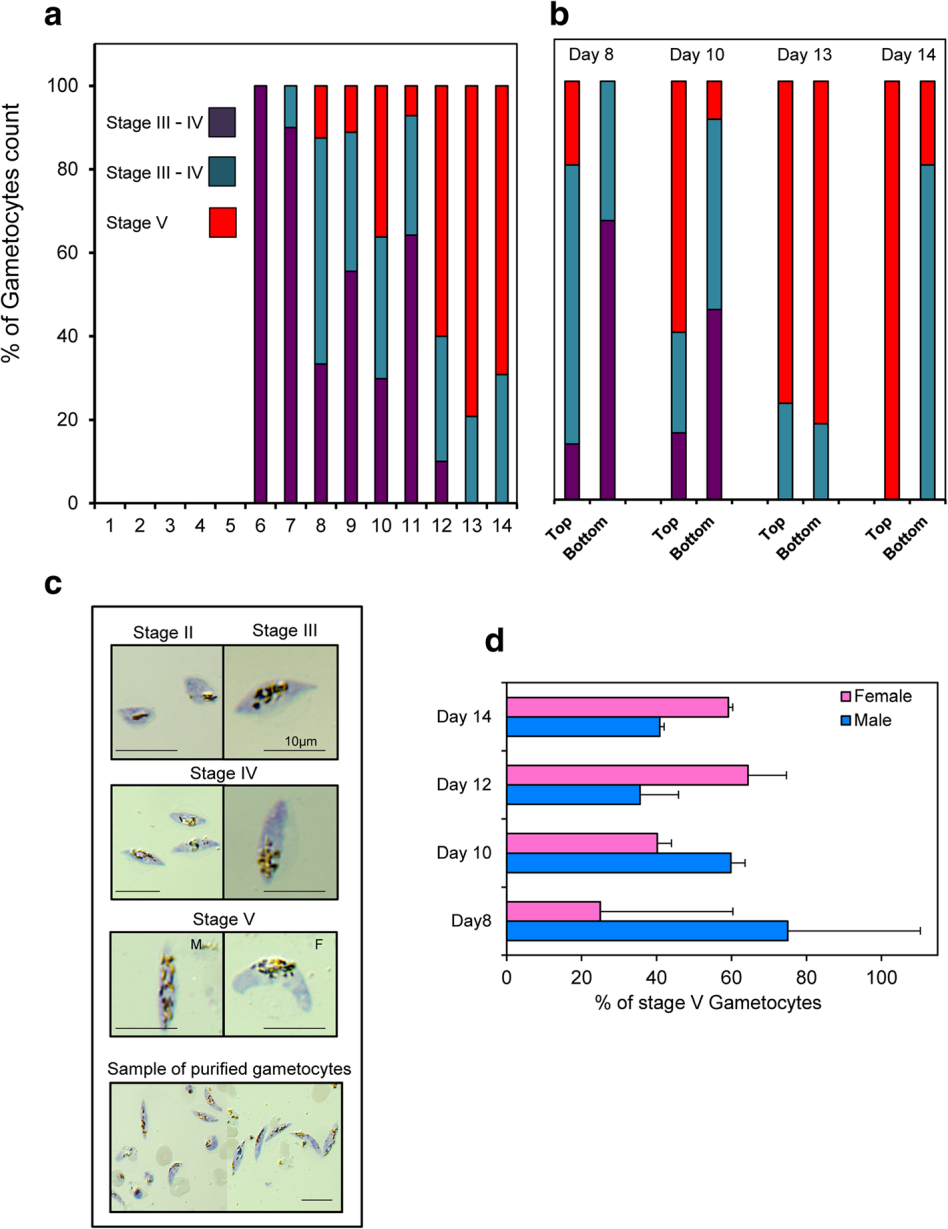
The development of *P. falciparum* gametocytes in the Wave Bioreactor. **a** Gametocyte maturity over the time. Each *bar* represents the percentage of a specific gametocyte stage over the total number of gametocytes (*averaged over the three experiments* shown in Fig. 2). **b** Comparison of Gametocyte maturity stages from samples collected in the Cellbag while rocking (“*bottom*”) or in the supernatant after the rocker was stopped for several hours (“*top*”). Bar represents 10 μm. **c** Pictures of Giemsa stained thin smears representing the different gametocyte maturity stages obtained during the culture in the Wave Bioreactor and sample of mature gametocytes harvested after purification. **d** Representation of sex ratio of mature gametocytes obtained by analysing thin smears prepared in three different experiments. Each bar corresponds to the percentage of male or female over the total number of mature stage V gametocytes

**Fig. 4:**
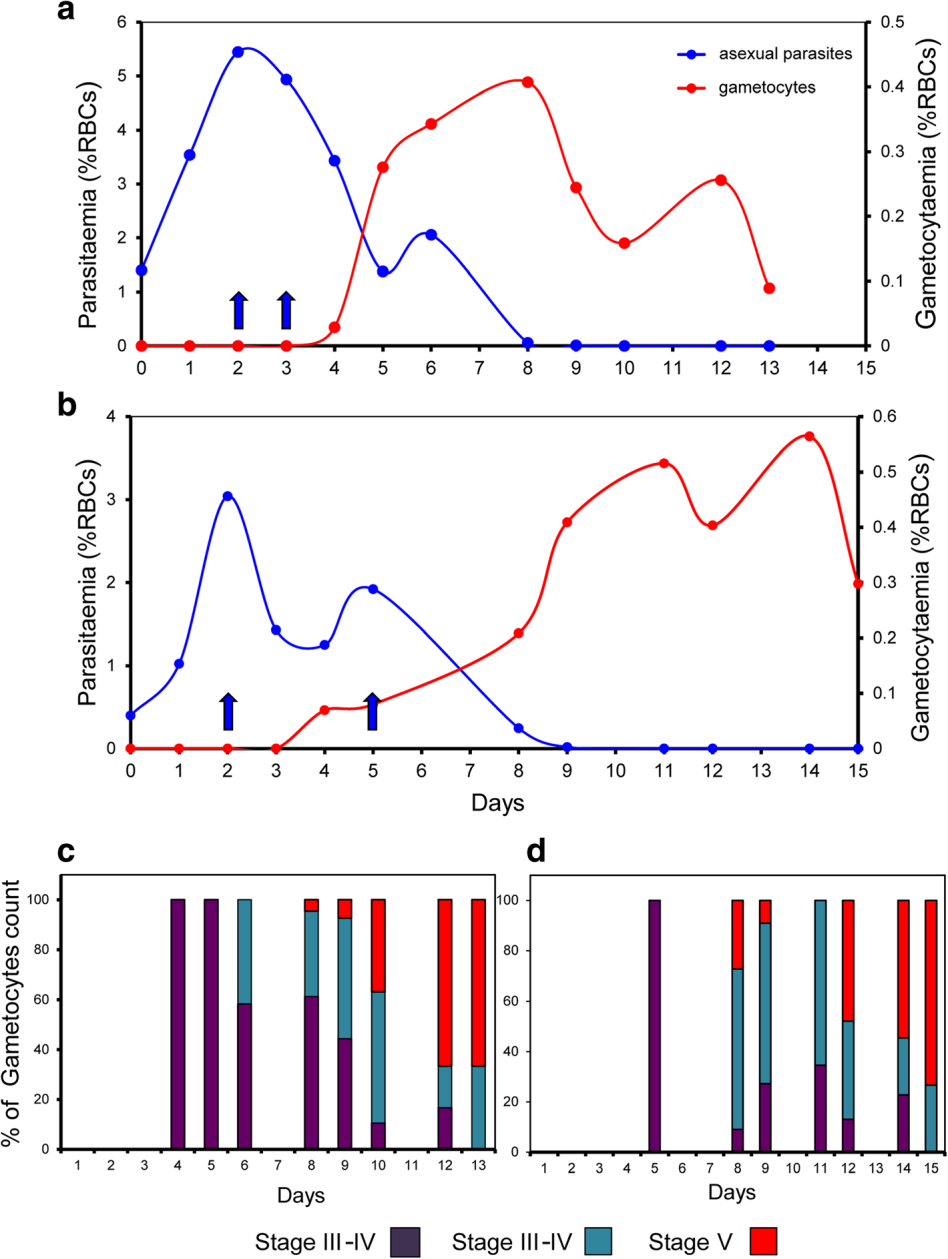
Induction of gametocytes in the Wave Bioreactor from synchronous *P. falciparum* blood stage cultures. **a**, **b** Time course of parasitaemia and gametocytaemia for two different experiments performed, respectively, at McGill university (Canada) and in QIMR (Australia), *arrows* represent the days when GlcNAc were added; cultures were initiated using synchronized ring-stage parasites. **c**, **d** Diagrams of stage development of gametocytes over the time for the corresponding experiments

N-acetylglucosamine (GlcNac), at a concentration of 50 mM, was previously shown to stop the multiplication of asexual parasites over a 72 h period without harm to gametocyte development [26]. In our system, several initial tests with asynchronous and synchronous cultures allowed us to determine that GlcNac at a concentration of 20 mM for 48 h successfully inhibited asexual parasite growth in the Cellbag (Fig. 1). GlcNac was added to the Cellbag 2 days after cultures were established when the parasitemia was between 2.5 and 3.5% for asynchronous culture and between 3 and 6% for synchronous culture. This timing was optimal as it allows the asexual parasitaemia to be sufficiently high to allow for the generation of large numbers of gametocytes, but not so high as to overly stress the culture (Figs. 2a and 4a, b). The parasitaemia dropped abruptly during the 48 h following the addition of GlcNac to the Cellbag, then the remaining number of asexual stage parasites slowly diminished before completely disappearing over the following five to 7 days (Fig. 2a). The same growth pattern was observed with synchronous cultures in the experiments performed in two different research laboratories (McGill University, Canada, and QIMR Berghofer Medical Research Institute, Australia) (Fig. 4a, b). The number of gametocytes started to rise soon after the addition of GlcNac, day 3 to 4 from the initiation of the culture, and developed to become mature stage V gametocytes over the following days (Figs. 2, 3 and 4).

### The development of *P. falciparum* gametocytes in large-scale culture

Sexual stage cultures produced in the Wave Bioreactor from asynchronous blood stage cultures were characterized by the occurrence of successive “waves” of gametocytes (Fig. 2b). The development of gametocytes initiated with cultures containing synchronous asexual stage parasites exhibited more synchronicity (Fig. 4a, b). We observed that RBCs containing mature gametocytes tend to float (Fig. 3b) which posed a problem when medium needed to be changed (it is necessary to slow down the rocking platform to allow erythrocytes to settle so that medium can be drawn from the top, Additional file 1: Figure S1). However, we discovered that it was not necessary to change the medium in the cultures after day 7 (Fig. 2). Indeed, from day 8 onwards the lactic acid level and pH of the culture medium remained stable, most likely because gametocytes consume far less glucose than do asexual blood stage parasites and therefore produce less lactic acid (see Fig. 2c).

Gametocyte maturation is divided into five developmental stages (stages I to V) as distinguished on Giemsa-stained smears (Fig. 3c). Indeed, stage I gametocytes are morphological identical to young asexual trophozoite and are very difficult to identify on Giemsa stained blood film [5]. Mature stage V gametocytes first appeared on day 8 of the culture in the Wave Bioreactor and the number progressively increased thereafter to day 15 (Figs. 3a and 4c, d). During the development of gametocytes to full maturity we observed a predominance of male gametocytes from day 8 to day 11+/−1 before the females became more abundant (Fig. 3d). The observation that male gametocytes reach maturity earlier, or have a shorter lifespan, than female gametocytes which results in a greater abundance of female gametocytes in the final culture has also been reported by others [30].

### Viability, infectivity and cryopreservation of gametocytes produced in the Wave Bioreactor

*Plasmodium falciparum* 3D7 gametocytes produced in the bioreactor were isolated using magnet-activated cell sorting (MACS) as previously described [4]. This represents a logistical bottleneck for large-scale purification since a 10 ml MACS column is recommended for the isolation of parasites from about 1 ml PCV of erythrocytes (in the Wave Bioreactor, we routinely have 25 to 30 ml PCV). Nevertheless, the isolation of gametocyte can be spread over several days; we recommend taking samples from the Cellbag from day 10 to day 13 (see below).

An alternative means of isolating gametocytes takes advantage of the fact that gametocytes are more buoyant than uninfected cells and tend to float. Therefore, when the rocking motion of the platform was slowed, uninfected erythrocytes settled to the bottom and the more buoyant gametocyte-infected RBCs remained at the top and could be drawn off via the exit port. These could then be further purified using a MACS column. In our studies, we could isolate up to 100 to 110 million gametocytes from a 1 l culture.

**Fig. 5:**
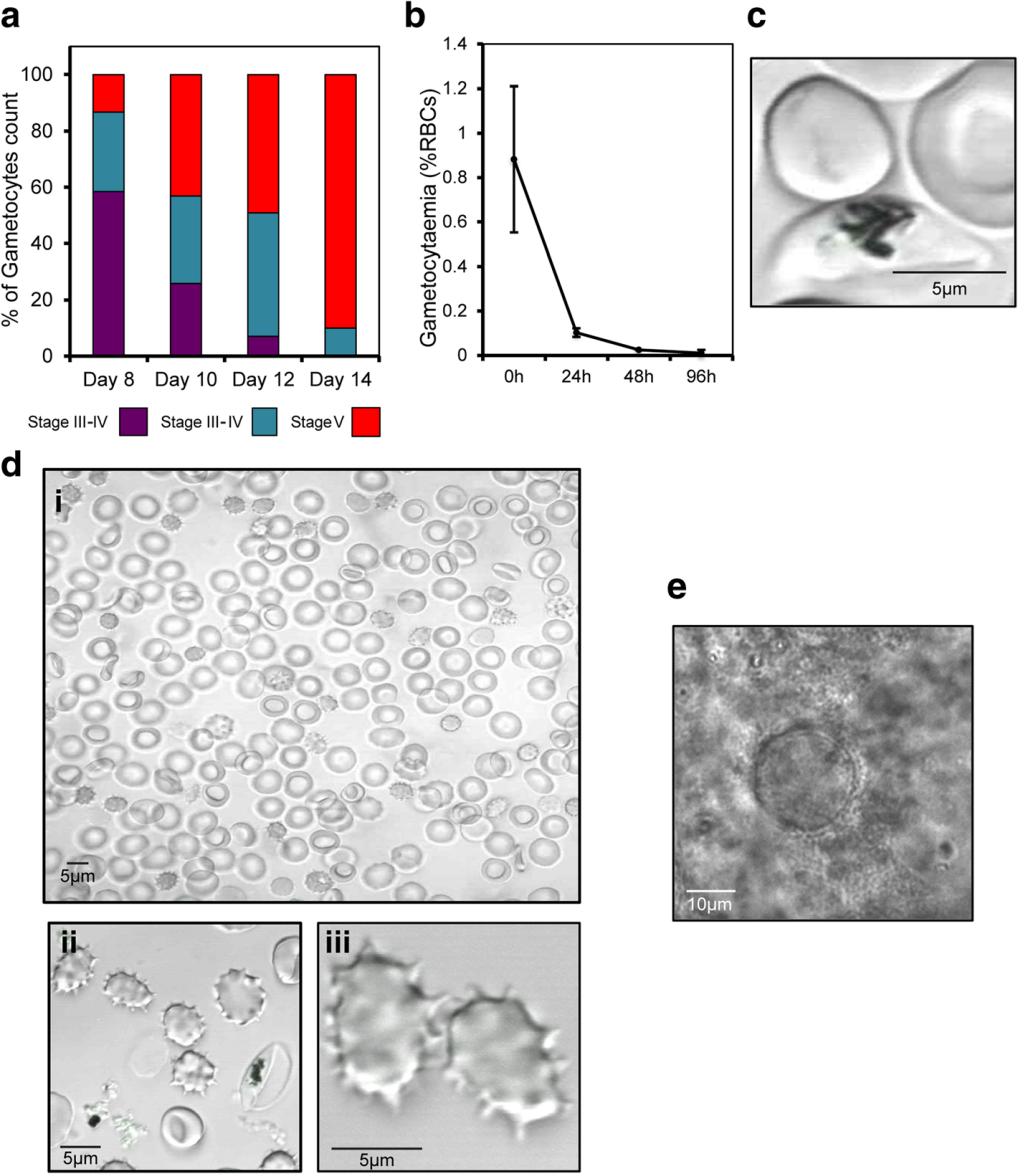
Assessment of cryopreserved malaria gametocytes. **a** *P. falciparum* gametocyte maturity stages obtained after thawing samples from wave Bioreactor frozen on different days. The diagram represents data. **b** Viability over the time of *P. falciparum* gametocytes after thawing. The *curve* represents mean values obtained after thawing in three independent experiments the samples collected in the Wave Bioreactor on day 10. The thawed cells were cultured at 37 °C during 4 days. **c** Photograph of a healthy mature gametocyte obtained immediately after thawing of frozen sample. Its represents DIC image of cell suspension observed with confocal microscope 60× objective. **d** Representative DIC images of *P. falciparum* gametocytes during exflagellation test performed after thawing of frozen samples taken at 10× objective to show abundance of exflagellating gametocytes (i), at 20× and (ii) and 60× (iii) to show detail of gametocytes with flagella (spike-like projections). **e** Development of midgut oocysts 10 days after mosquito membrane feeding of gametocyte frozen sample from Wave Bioreactor. Picture is at 40× magnification

A methodology for the cryopreservation of early stage *P. falciparum* gametocytes in Glycerolyte 57 was described by Keister and Kaslow [31]. This was subsequently shown to also be suitable for the cryopreservation of stage IV and V gametocytes by Peatey et al. [14]. We derived these reported methods to successfully cryopreserve and retrieve gametocytes at all stages of development (Fig. 5a). Gametocytes isolated from the Cellbag were frozen on day 10–13; these days were judged to be the best time to prepare mature stage V gametocytes with a good ratio of male and female forms (Figs. 3a, d and 4c, d). Samples of the cryopreserved parasites were thawed and examined in exflagellation tests. The exflagellation of male gametocytes post-thawing was observed after incubation at 25 °C for 30 to 45 min in the presence of xanthurenic acid (Fig. 5d).

Tests were also performed to determine if resurrected parasite could infect mosquitoes. These tests were carried out on the same day as thawing since a sharp decline in viability was observed when gametocytes were cultured at 37 °C (a reduction of > 50% during the first 24 h, Fig. 5b). *P. falciparum* gametocytes remained infectious after cryopreservation as we observed 2–4 oocysts in 6 of 25 laboratory-reared *An. stephensi* mosquitoes after membrane feeding (Fig. 5e). However, no sporozoites could be detected at 16–20 days after membrane feeding due to the small quantity of mosquitoes dissected and low infection rate.

## Discussion

Here we describe methods for large scale in vitro culture of *P. falciparum* blood stages in a Wave Bioreactor system. Because parasites cultured in this system are kept in suspension by the slow rocking of the media, nutrients are equally available to all cells, metabolites (including lactic acid) are quickly dispersed and the pH throughout the culture is balanced. As a result, malaria parasites exhibit improved grow rates than in static cultures, parasite cell synchronicity is preserved and the number of multiple-infected erythrocytes is much reduced [27]. The addition of a reservoir for holding medium has made the operation of the system more convenient for changing medium and because the culture remains continuously on the heated rocking platform parasites do not experience temperature or gas fluctuations. Moreover, since the system is completely closed, the risk of contamination from the environment is minimal.

We describe a straightforward method to produce mature gametocytes of *P. falciparum* in the Wave Bioreactor, a procedure that requires a continuous and stable culture for 10 to 16 days. The low-shear rocking, constant temperature, good gas exchange, and regular medium exchange in the bioreactor are more conducive for the development of gametocytes than in static cultures. We can now reproducibly generate and purify approximately 100 million gametocytes from a 1 l culture. Samples of parasites of sufficient quantities can be readily taken at any time-points during the culture to allow stage-specific studies and batches of maturing gametocytes can be collected from the same Cellbag culture over several days.

Exflagellation tests verified that gametocytes produced in our system are viable, and can be stored frozen in small aliquots and re-vitalized in vitro when needed. We have described protocols for the freezing and thawing of gametocytes from stage III to V, although we found that after thawing gametocyte viability drops abruptly within the first 24 h of culture. Most importantly, our results provide clear evidence that mature gametocytes thawed from storage can exflagellate on the same day as thawing.

The production of gametocytes in large volumes using the Wave Bioreactor saves labour, time and costs compared to using multiple flasks, and is less dependent on the operator’s expertise in malaria cell culture. The large-scale culture offers the potential for continuous, potentially automated, sampling of mature gametocytes from the same culture for studies of stage development, organelle isolation and other analyses requiring larger quantities of parasite products (e.g. antigens for transmission blocking vaccine, studying of gametocytes commitment and development, proteomics etc.). Greater efficiency and experimental reproducibility can be achieved since multiple aliquots of gametocytes can be frozen from the same culture preparation (potentially hundreds of identical aliquots) for use in controlled standardized assays and other assays requiring reference parasites. For example, thousands of anti-gametocyte drug tests could be potentially performed using parasites from a 1 l culture in high throughput drug discovery assays.

Despite the recent advances in the development of molecular vaccines for malaria whole-cell malaria vaccines could still hold great promise particularly because they can overcome issues of antigenic variation and polymorphism [7–10]. However, one of the major drawbacks is the lack of large-scale parasite production methods. The quantities of blood-stage malaria parasites (asexuals and gametocytes) that can be obtained from cultures in the Wave Bioreactor now make the manufacture of whole-cell vaccines under GMP-compliant procedures feasible. As well, if optimised, infection of mosquitoes from frozen gametocytes could facilitate the production of large quantity of sporozoites for vaccine purposes.

## Conclusion

The largescale production of asexual [27] and gametocyte stages of *P. falciparum* in the Wave Bioreactor should facilitate cellular, developmental and molecular studies of this major human parasites. The quantities of parasites that can be readily obtained under GMP-conditions could also simplify the pipeline for high-throughput screening for new anti-malarial drugs and, possibly, the development of whole-cell asexual, gametocyte or sporozoite-based vaccines. Furthermore, since we have shown that cryopreserved aliquots of gametocytes taken from these large-scale cultures can be transported to other laboratories on dry ice for functional studies it is possibility that a single laboratory could act as a service provider of frozen gametocytes.

## Additional files

Additional file 1: Figure S1. Protocol to produce asexual blood stages of *P. falciparum* in a Wave Bioreactor. (PPTX 29 kb)

Additional file 2: Figure S2. The proliferation of asexual blood stage malaria parasite in the Wave Bioreactor. **a** Parasitemia curves of *Plasmodium falciparum 3D7* and *FCR3* strains cultured in 3–5 days; **b** Time course of glucose consumption and lactate production; **c** pH fluctuations over period of culture. (PPTX 23 kb)

## Abbreviations

GlcNac: N-acetylglucosamine
GMP: Good manufacturing process
MACS: Magnet-activated cell sorting
MFA: Membrane feeding assays
PCV: Packed cell volume
RBC: Red blood cell
RPMI 1640: Royal Park Memorial Institute 1640
XA: Xanthurenic acid
