# Supplementary figures and images for: The development of sexual stage malaria gametocytes in a Wave Bioreactor
Source: Parasit Vectors. 2017 May 2;10:216. doi: 10.1186/s13071-017-2155-z (PMC5414375; doi:10.1186/s13071-017-2155-z)

## Slide 1
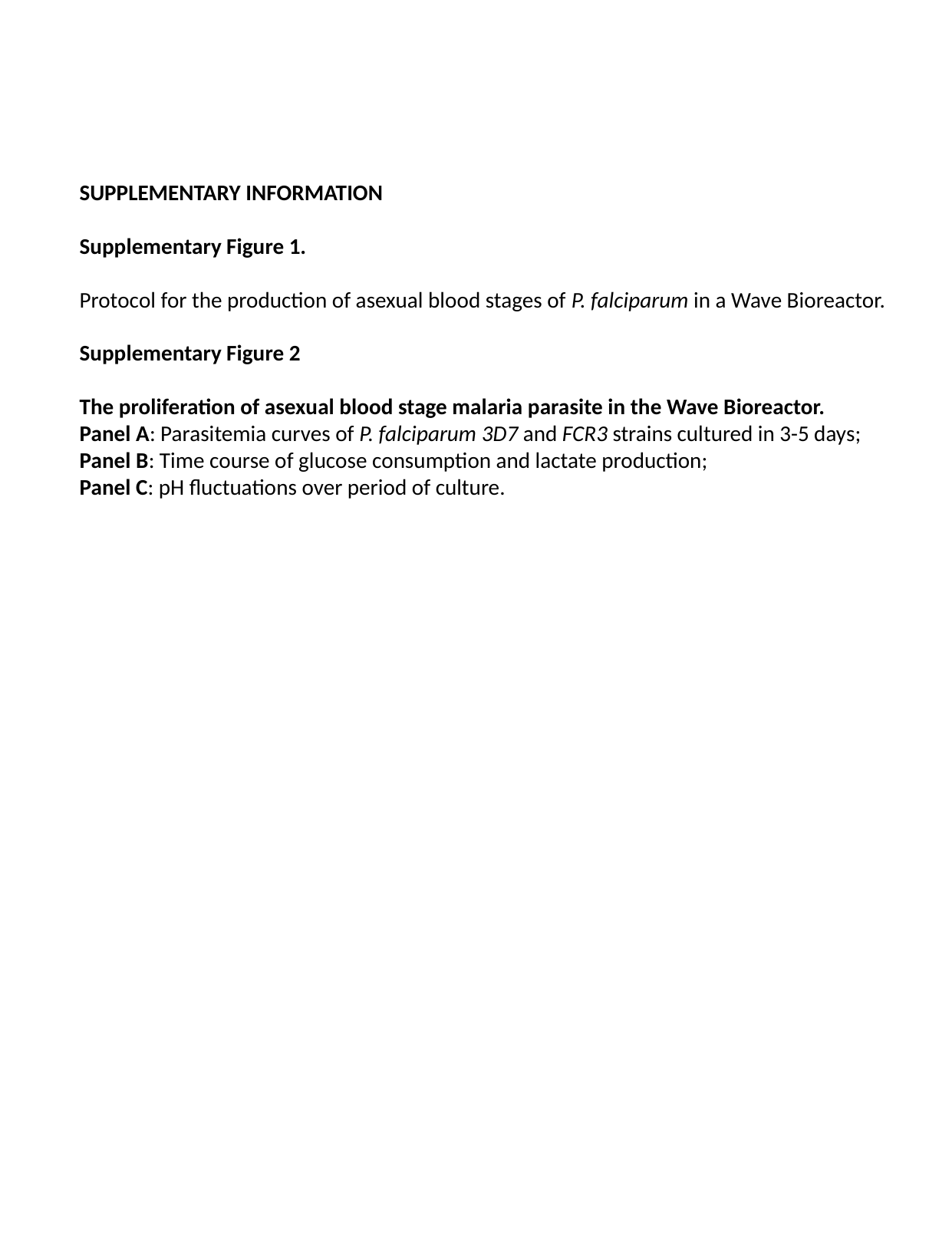

## Slide 2
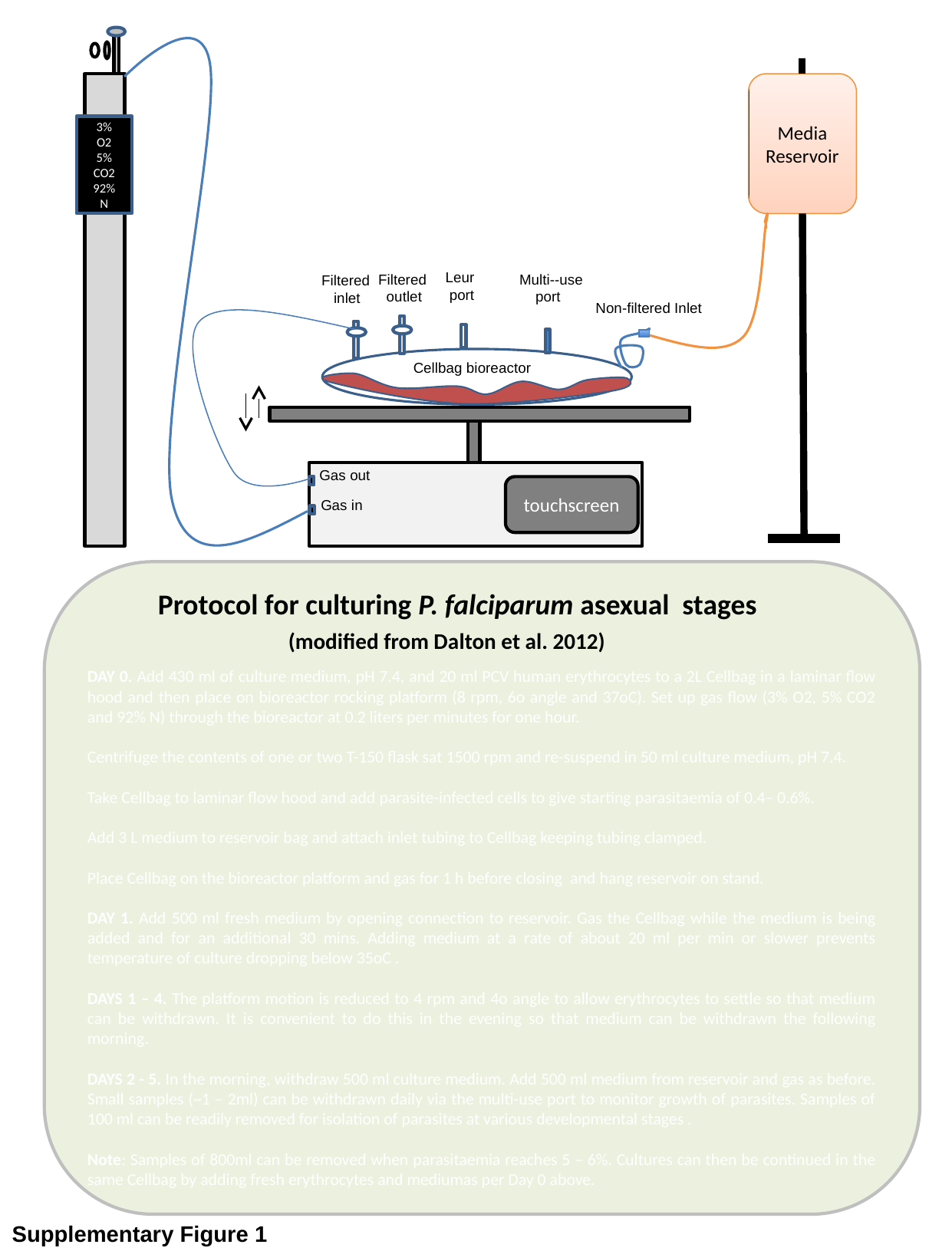

Supplement: Supplementary file 1 — Protocol to produce asexual blood stages of P. falciparum in a Wave Bioreactor. (PPTX 29 kb) [file 13071_2017_2155_MOESM1_ESM.pptx]

## Slide 1
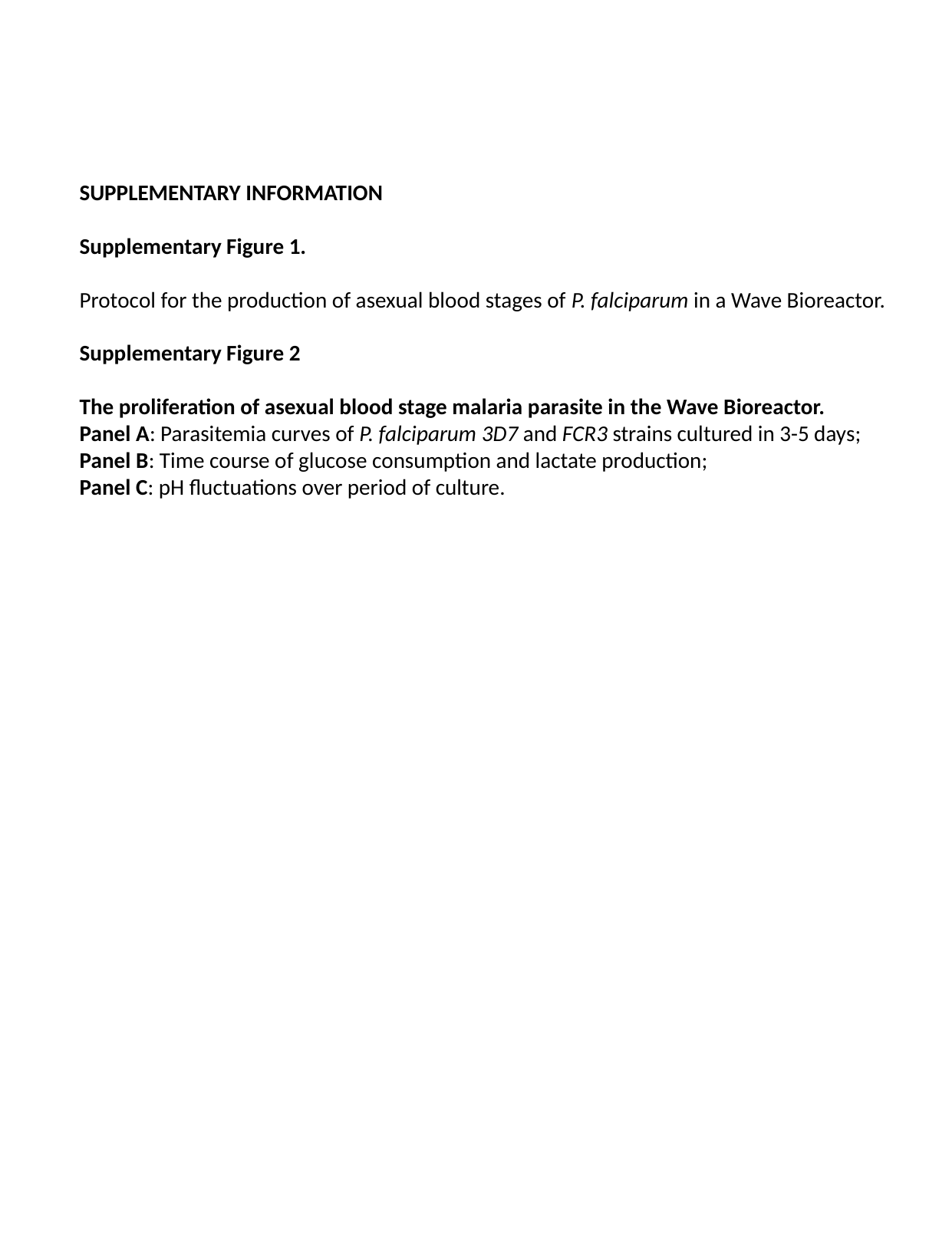

## Slide 2
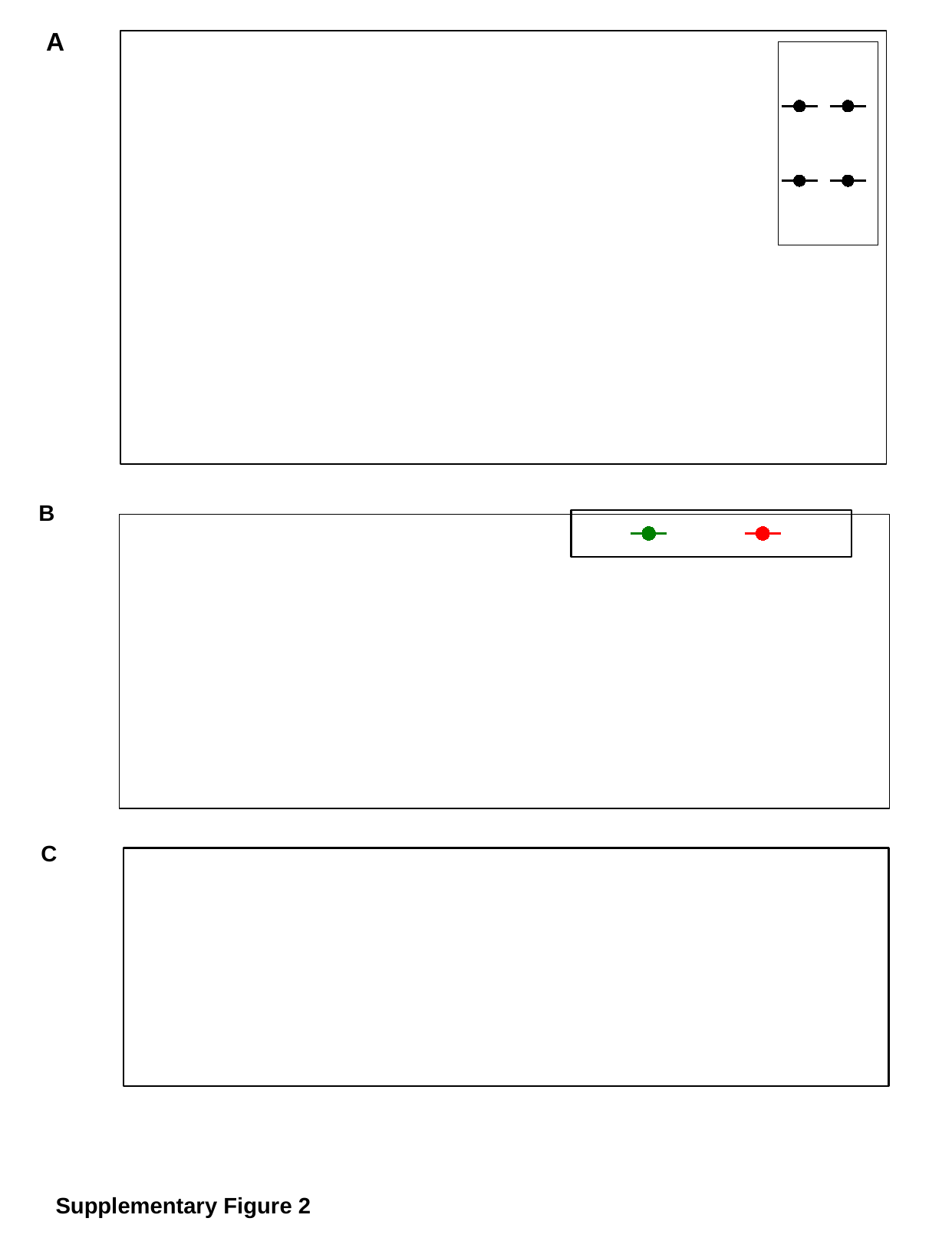

Supplement: Supplementary file 2 — The proliferation of asexual blood stage malaria parasite in the Wave Bioreactor. a Parasitemia curves of Plasmodium falciparum 3D7 and FCR3 strains cultured in 3–5 days; b Time course of glucose consumption and lactate production; c pH fluctuations over period of culture. (PPTX 23 kb) [file 13071_2017_2155_MOESM2_ESM.pptx]
